# Items

**Published:** 1881-12

**Authors:** 


### Items.

---

THE *New York Medical Record* makes the assertion, based upon the report of the board of censors of the medical society of the county of New York, that the medical law of 1880, the registration act, is a failure in that state.

This conclusion, reluctantly arrived at by the committee, is the result of abundant experience in the police courts and before the grand jury, obtained in the prosecution of individuals who have been guilty of infringing or evading the provisions of this law.

The district attorney has expressed the opinion that false swearing, under the act, does not constitute perjury, according to a strict construction of the meaning of the term, and cannot be punished as such.

If this view of the case is sustained by the courts, the law becomes inoperative and virtually dead.

It is fortunate that this obstacle to the enforcement of a similar law in this state does not exist. The language of the acts, on this point, is different.

---

THE Governor, in conformity with the requirements of the new pharmacy law, has appointed the following gentlemen to constitute the board of pharmaceutic examiners. Messrs. Edward Barry, Augusta; Isidore Zacharias, Columbus; J. S. Pemberton, Atlanta; John Ingalls, Macon, and Oceola Butler, Savannah. At the meeting for organization in this city, on November, 9th, Mr. Barry was elected chairman and Mr. Zacharias was elected secretary.

The Board adjourned to meet for the examination of druggists and the granting of licenses, in Atlanta, on the thirteenth day of December.

---

WE are indebted to Dr. J. H. Branham of Baltimore, for the report of the proceedings of the Baltimore Medical and Surgical Society at a late meeting.

Dr. Branham, is assistant demonstrator of anatomy in the College of Physicians and Surgeons in that city, and official reporter for the society. He is a native of Georgia and well represents the state of his birth in his adopted home.

We trust that we shall be frequently favored, through the courtesy of Dr. Branham, with similar reports.

---

IN justice to the publishers of the American edition of Holmes system of surgery it is proper to state, on the authority of *Gaillard's Journal*, that the English publishers, who are also the proprietors of the work, were informed of the intentions of Messrs. Lea & Company. It is said, too, that the American publishers offered to purchase the wood-cuts used in the English edition, and furthermore tendered a sum of money in consideration of the republication.

---

THE registration of medical practitioners is fairly under way in this—Fulton—county.

The clerk of the superior court has provided, not only a book, which the law requires, neatly and appropriately ruled, but, for convenience, he supplies printed blanks for the affidavits demanded by the registration act, which, after being properly filled, are carefully filed, and made a part of the records of his office.

---

WOULD it not be well for druggists who are in the habit of dispensing medical advice—that is, prescribing for symptoms and then filling their own prescriptions—to read attentively the act to regulate the practice of medicine in Georgia? Under the law every one must register his lawful authority, or not undertake to practice medicine. We offer this suggestion *gratis.*

---

DR. C. A. BRYCE, editor of the *Southern Clinic*, of Richmond, Va., became involved in some differences with the State Medical Society, because of charges made by him against the publishing committee. A large part of his October issue is devoted to the proceedings of the society relating to the case. The matter seems to have been finally adjusted.

---

TONER ON TRAINING.—We have received a newspaper clipping, with the above heading, embracing an address by J. M. Toner, M.D., Washington, D. C., delivered on the occasion of the opening of the Washington Training School for Nurses. He was preceded by Dr. Beale, of the faculty, in an address on The Requirements of a Nurse.

---

DR. ALFRED CHARLES HOLT, a native of Augusta, Ga., whose home was in New Orleans, died October 5th. He was one of the administrators of the Charity Hospital, and Professor of Clinical Medicine in the New Orleans School of Medicine. He took a prominent part in redeeming Louisiana from carpet-bag rule.

---

"UNCLE WILLIAM'S PILLS."—A little girl came into a drug store and asked for five cents worth of "Uncle William's pills." The druggist could not make it out, so he sent her away; she returned soon afterwards, and said, "Mother said 'Aunty Billy's,' but I thought it couldn't be right—*Boston Jouanal of Chemistry.*

---

IT will be interesting to observe the effect of the medical practice act upon the proprietors and vendors of various well known nostrums in this state.

It remains to be seen just how far and how effectually the law will apply to such cases. Let us hope for the best.

---

VISITING PHYSICIANS.—We have had the pleasure during the past week or two, of meeting many physicians of this and adjoining States, who are attracted here by the Cotton Exposition. We hope to meet many more. A physician never gets more recreation than he deserves.

---

THE lowest price of opium in the United States for the ten years ending 1880 was in 1878, when it sold for $4.35 per pound. This year it has been still lower, a lot in New York having sought a purchaser at $4.00.—*Pacific Med. and Surg. Journal*

---

ANOTHER medical college, rumor says, is to be established in Louisville. The lectures, it seems, are to be given in the summer season. The faculty is to be composed, in part at least, of members of some of the schools already established there.

---

THE NATIONAL ECLECTIC SOCIETY, at its meeting in St. Louis, placed on probation, for one year, the Eclectic Colleges at Atlanta and Indianapolis, on account of arrant quackery among the professors.—*Pacific Med. and Surg. Journal.*

A NEW INFECTIOUS DISEASE.—The *Michigan Medical News* quotes from a local paper the following : " There were fifteen pairs of twins born in Port Huron during the present year, and hundreds of families who have been exposed are anxious lest the disease be contagious.— *Chicago Med. Review.*

---

WE have a few more copies of the November number of the REGISTER containing the medical practice act, the law governing the granting of diplomas, and the pharmacy law, which will be sent to any address on receipt of 25 cents.

---

NEW ENGLAND MEDICAL MONTHLY.—We have received the first issue of this journal, edited by W. C. Wile, M.D., Sandy Hook, Conn. A short, business-like salutatory is presented. The editing and publishing are well done.

---

MEDICAL SOCIETIES.—The profession of this city seems to have fallen into a state of lethargy in regard to medical societies. We are expecting, though, that the period of reaction will soon set in.

---

IF the editor of the *St. Louis Courier of Medicine* will refer to his November number, he will find that he does not correctly announce the name of the ATLANTA MEDICAL REGISTER.

---

AT the fifth annual commencement of the Nurses' Training School, on Blackwell's Island, there were seventeen graduates. Dr. F. N. Otis addressed the class.

---

REGISTER.—Not one-third of the physicians of the city have yet registered.

